# Longitudinal mediation in the PACE randomised clinical trial of rehabilitative treatments for chronic fatigue syndrome: modelling and design considerations

**DOI:** 10.1186/1745-6215-16-S2-O43

**Published:** 2015-11-16

**Authors:** Kimberley Goldsmith, Trudie Chalder, Peter White, Michael Sharpe, Andrew Pickles

**Affiliations:** 1 Institute of Psychiatry, Psychology & Neuroscience, King's College London, London, UK; 2 Centre for Psychiatry, Wolfson Institute of Preventive Medicine, Barts and the London School of Medicine, Queen Mary University, London, UK; 3 Psychological Medicine Research, Department of Psychiatry, University of Oxford, Oxford, UK

## Background

Clinical trials require large monetary and time commitments and should provide information on both whether and how treatments work. Treatment mechanisms can be studied using mediation analysis, allowing refinement of treatments. Mediation studies often use only single contemporaneous measures of mediator and outcome limiting the conclusions that can be drawn. Longitudinally measured mediators and outcomes, such as those in the Pacing, Graded Activity, and Cognitive Behaviour Therapy: A Randomised Evaluation trial (PACE, ISRCTN 54285094) allow for more realistic estimates of mediated effects.

## Methods

Autoregressive models accounting for measurement error were used to study treatment effect mediation of cognitive behaviour therapy (CBT) and graded exercise therapy (GET) in PACE. Fear avoidance and physical function were used as example mediator and outcome; these were measured at baseline and three times post-randomisation as part of the trial design. Model fit criteria, Wald tests and comparisons of parameter estimates were used.

## Results

Longitudinal SEM were more flexible and gave what were likely more plausible estimates of mediated effects. Constancy of mediator - outcome effects over time and across treatment groups increased precision. For CBT and GET, 46% and 53% of the overall effect were mediated through fear avoidance.

## Conclusions

Trials should be designed to include multiple measurements of mediators and outcomes so that more realistic mediation models can be used. Longitudinal models may have more power to detect mediated effects. Approximately half of the effect of each of CBT and GET were on physical function was mediated through reducing avoidance of fearful situations.

